# Alternative Functions of *Arabidopsis YELLOW STRIPE-LIKE3:* From Metal Translocation to Pathogen Defense

**DOI:** 10.1371/journal.pone.0098008

**Published:** 2014-05-20

**Authors:** Chyi-chuann Chen, Wei-Fu Chien, Nai-Chun Lin, Kuo-Chen Yeh

**Affiliations:** 1 Agricultural Biotechnology Research Center, Academia Sinica, Taipei, Taiwan; 2 Department of Agricultural Chemistry, National Taiwan University, Taipei, Taiwan; UMBC, United States of America

## Abstract

YELLOW STRIPE-LIKE1 (YSL1) and YSL3 are involved in iron (Fe) and copper (Cu) translocation. Previously, we reported that upregulation of *YSL1* and *YSL3* under excess Cu caused high accumulation of Cu in the *siz1* mutant, impaired in small ubiquitin-like modifier (SUMO) E3 ligase. Interestingly, the *siz1* mutant contains high levels of salicylic acid (SA), involved in plant defense against biotrophic pathogens. In this study, we found that *YSL1* and *YSL3* were upregulated by SA. SA-regulated *YSL3* but not *YSL1* depended on *NONEXPRESSOR OF PR1* (*NPR1*). Susceptibility to the pathogen *Pseudomonas syringe* pv. tomato (*Pst*) DC3000 was greater for *ysl3* than the wild type. Also, during *Pst* DC3000 infection, *YSL3* was positively regulated by SA signaling through *NPR1* and the upregulation was enhanced in the *coi1* mutant that defective in the jasmonic acid (JA) receptor, *CORONATINE INSENSITIVE1*. This line of evidence indicates that the regulation of *YSL3* is downstream of SA signaling and interplays with JA signaling for involvement in pathogen-induced defense. We provide new insights into the biological function of the metal transporter *YSL3* in plant pathogen defense.

## Introduction

Plant species have 2 classes of mechanisms for acquiring iron (Fe): Strategy I is used by nongraminaceous plants to reduce ferric chelates at the root surface and absorb the generated ferrous ions across the root plasma membrane by iron transporters; strategy II is a chelation strategy used by graminaceous plants for primary acquisition of Fe [1]. Strategy II plants secrete phytosiderophores (PSs), compounds of the mugineic acid family, which are hexadentate metal chelators with high affinity for ferric Fe [2]. PSs are derivatives of the non-proteinogenic amino acid nicotianamine (NA), which also functions as a transition metal chelator in plants [3], [4]. In grasses, ferric Fe–PS complexes in the rhizosphere are taken up into root cells through the action of YELLOW STRIPE1 (YS1) transporter [5]–[9].

The *YELLOW STRIPE-LIKE* (*YSL*) family in Arabidopsis was identified by sequence similarity to maize *YS1* (*ZmYS1*), which takes up ferric Fe–PS complexes and belongs to the oligopeptide transporter family [7], [10], [11]. Multiple *YSL* genes are found in diverse plant species, including monocots, dicots, gymnosperms, ferns and mosses [5], [7], [10], [12]–[18]. The Arabidopsis *YSL* family has 8 members [7]. They play important roles in plant Fe homeostasis. Some *YSL* genes may have a role in metal remobilization from senescent leaves, in the development of reproductive organs and as transporters in seed formation, and in long-distance transport of metals complexed with NA [13], [19], [20].

Arabidopsis *YSL1*, *YSL2* and *YSL3* are located in the plasma membrane and expressed in the vascular bundle parenchyma. Their functions likely mediate the remobilization of Fe, Zn, and Cu in the form of metal–NA chelates from senescent leaves and the loading of these metals into inflorescences and seeds [16], [21]–[24]. Recently, YSL4 and YSL6, reported as potential H^+^/metal–NA co-transporters, were found localized in vacuole membranes and internal membranes resembling endoplasmic reticulum [25], [26]. YSL6 was also detected in the chloroplast envelope. Fe is trapped in the chloroplasts of *ysl4ysl*6 double mutants, which suggests a fundamental role for *YSL4* and *YSL6* in managing chloroplastic Fe content [27].

The expression of *YSL2* is induced in the presence of Fe and Cu and repressed strongly by Zn deficiency and mildly by Cu excess [16], [22]. Under Cu deficiency, among the Arabidopsis *YSL* genes, *YSL2* expression is upregulated by *SQUAMOSA PROMOTER BINDING PROTEIN-LIKE7* (*SPL7*), and induction of *YSL3* expression partially depends on *SPL7* function [28], [29]. Moreover, the transcriptional regulation of *YSL1* and *YSL3* is repressed by Fe deficiency and induced by Fe excess [16], [21]–[23]. Our previous work revealed that both *YSL1* and *YSL3* were downregulated by SIZ1-dependent SUMOylation that induced by excess Cu in Arabidopsis (Chen et al., 2011). The expression of *YSL1* and *YSL3* were dramatically higher in the *siz1* mutant than in wild type.

Interestingly, the *siz1* mutant showed high levels of salicylic acid (SA) and SA glucoside [30]. Elevated SA level in *siz1* induced the expression of pathogenesis-related (PR) genes and increased the resistance against the bacterial pathogen *Pseudomonas syringae* pv. *tomato* (*Pst*) DC3000 [31]. We hypothesized that the *YSL1* and *YSL3* could be regulated by SA and probably involved in the pathogen defense. In this study, we found that the expression of *YSL3* was induced by SA in an *NONEXPRESSOR OF PR1* (*NPR1*)-dependent pathway and a biological role of *YSL3* in the innate immunity of plants was illuminated.

## Materials and Methods

### Plant Materials and Growth Conditions

The *Arabidopsis thaliana* wild type (Col-0 and Col-6), *ysl1* (*ysl1-2*; SALK_034534), *ysl3* (*ysl3-1*; SALK_064683C), (*ysl3-2*; SALK_045218), *ysl1ysl3* (*ysl1-2ysl3-1*), *npr1* (*npr1-1*) and *coi1* (*coi1-16*) were described previously [32]–[34]. Seeds were surface-sterilized and grown on half-strength MS medium (1/2×MS salt, pH 5.7, 1% sucrose and 0.35% phytagel). Soil-grown plants were grown in pots containing a mixture of organic substrate, vermiculite and mica sheets (9∶1∶1 v/v). All plants were grown under 16-h light (70 µmol m^−2^s^−1^)/8-h dark, 22°C. For SA treatment, 12-day-old plants were transferred to 1/2 MS medium containing SA.

### RNA Isolation and Quantitative Real-time RT-PCR (qPCR)

Extraction of total RNA and qPCR were performed as described (Chen et al., 2011). Frozen shoot and root tissues (approximately 100 mg) were ground in liquid nitrogen with use of a tissue homogenizer (SH-48, J&H Technology Co.), to which 1 mL of TRIzol reagent was immediately added for RNA isolation. The concentration of the RNA was determined spectrophotometrically at 260 nm (Nanodrop, Isogen Life Science). Subsequently, 2 µg RNA was treated with RQ1 RNase-free DNase (Promega), and the reaction buffer was replaced with 5× first-strand RT buffer (Invitrogen). The cDNA was synthesized by use of SuperScript III reverse transcriptase (Invitrogen). qPCR analyses involved use of the KAPA SYBR FAST qPCR kit (Kapa Biosystems). The expression of *ACTIN2* (*ACT2*) was an internal control. The primers of qPCR are for *YSL1* 5′-TCCCAATGTGGTTCGCAGTTT-3′ (forward) and 5′-TTGAGACCGCAGCGAATGTA-3′ (reverse); *YSL3* 5′-GTGGCGGCAAATCTCGTTA-3′ (forward) and 5′-CCATCGGTAATGGAACCCAAT-3′ (reverse); *SID2* 5′-ATGCGGGACCTATTGGATTTT-3′ (forward) and 5′-TCTGATCCCGACTGCAAATTC-3′ (reverse); *PDF1.2* 5′-TTTGCTTCCATCATCACCCTTA-3′ (forward) and 5′-GCGTCGAAAGCAGCAAAGA-3′ (reverse); *PR1* 5′-GTCTCCGCCGTGAACATGT-3′ (forward) and 5′-CGTGTTCGCAGCGTAGTTGT-3′ (reverse); *ACT2* 5′-AGGTCCAGGAATCGTTCACAGA-3′ (forward) and 5′-CCCCAGCTTTTTAAGCCTTTGA-3′ (reverse). Testing of the efficiency of primers was based on the manufacturer's instructions (Applied Biosystems).

### Pathogenicity Assay of *Pseudomonas syringae*

Before infection, *Pseudomonas syringae* pv. tomato (*Pst*) DC3000 was grown in King's B (KB) medium [35] supplemented with 50 µg/mL rifampicin at 28°C. After overnight cultivation, a bacterial suspension was prepared in 10 mM MgCl_2_ with 0.02% Silwet L-77 to OD_600_ 0.05. Four-week-old Arabidopsis seedlings were inoculated by spraying the leaves with *Pst* DC3000 suspension until runoff was imminent and leaf surfaces appeared evenly wet [36]. Disease development was monitored every day and the bacterial population *in planta* was determined 3 days after inoculation as described [37]. In brief, 3 leaf discs were collected from plants in each pot by use of a 0.6 cm-diameter cork borer and homogenized in 10 mM MgCl_2_ by use of a plastic pestle. Serial dilutions of each sample were plated on selective KB medium agar supplemented with rifampicin and incubated at 28°C for 2 days, then the number of rifampicin-resistant colonies were counted for calculating colony-forming units per centimeter squared of infected leaf tissue. For analysis of gene expression, leaf samples with pathogen infection were harvested at different days post-inoculation (0, 1, 2, 3 dpi) and immediately frozen in liquid N_2_ and stored at −80°C.

### Statistical Analysis

Two-sample *t* test was used to examine statistical difference between the control and treatments. P<0.05 was considered statistically significant.

### Accession Numbers

Sequence data from this article can be found in the Arabidopsis Genome Initiative or GenBank/EMBL databases under the following accession numbers: *YSL1* (At4g24120), *YSL3* (At5g5355*0*), *SID2* (At1g14710), *PDF1.2* (At5g44420), *PR1* (At1g24610) and *ACT2* (At3g18780).

## Results and Discussion

### *YSL3* is positively regulated by SA signaling through *NPR1*

**Figure 1:**
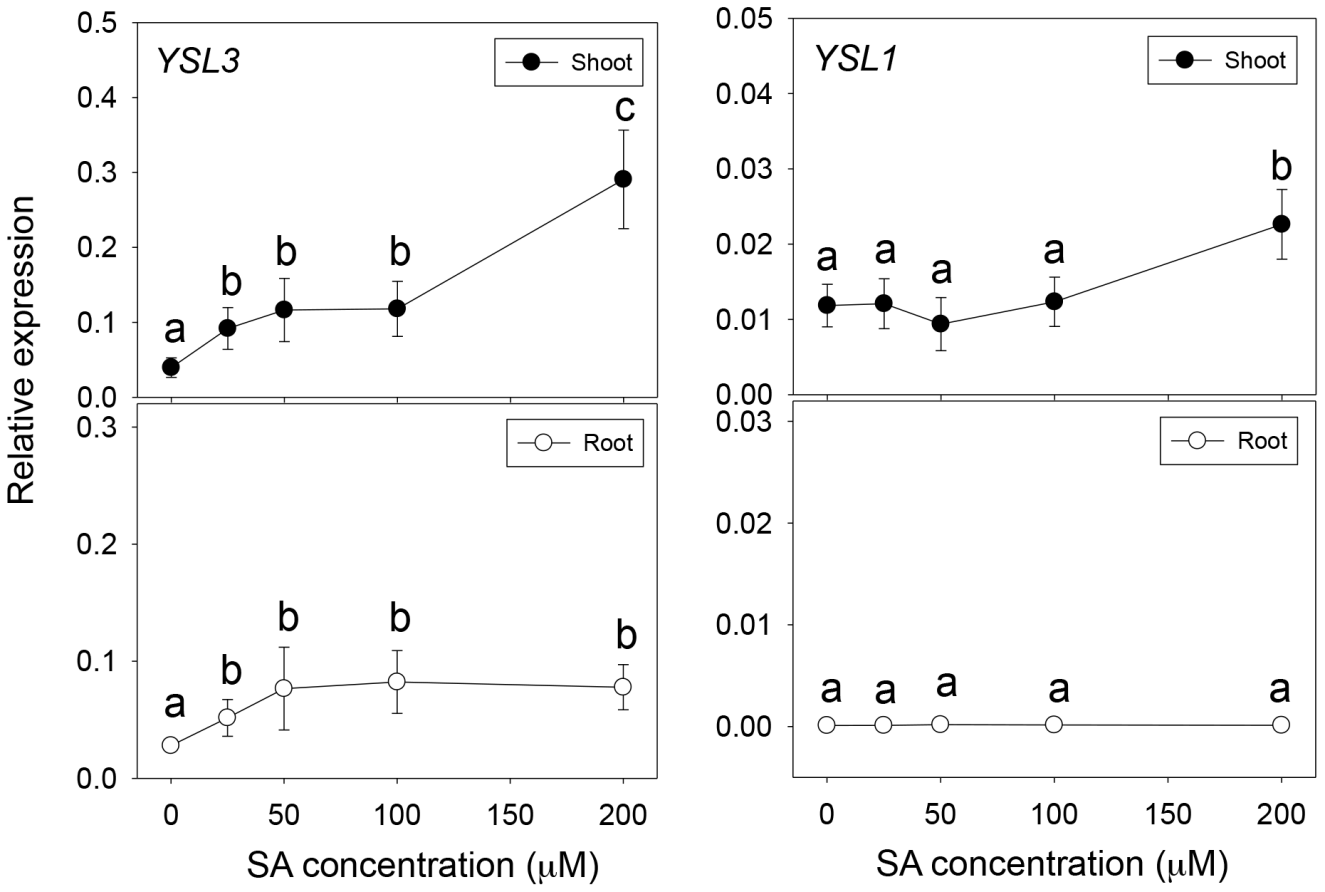
Dose effect of salicylic acid (SA) on the expression of *YSL1* and *YSL3* in shoots and roots of *Arabidopsis thaliana* ecotype Col-0. Twelve-day-old plants were treated with SA (0, 25, 50, 100 and 200 µM) for 24 h. Quantitative real-time RT-PCR (qPCR) analysis of expression of *YSL1* and *YSL3* in shoots and roots relative to that of *ACT2*. Data are mean±SD from 9 samples from 3 biological replicates. Different letters indicate significant difference at p<0.05.

**Figure 2:**
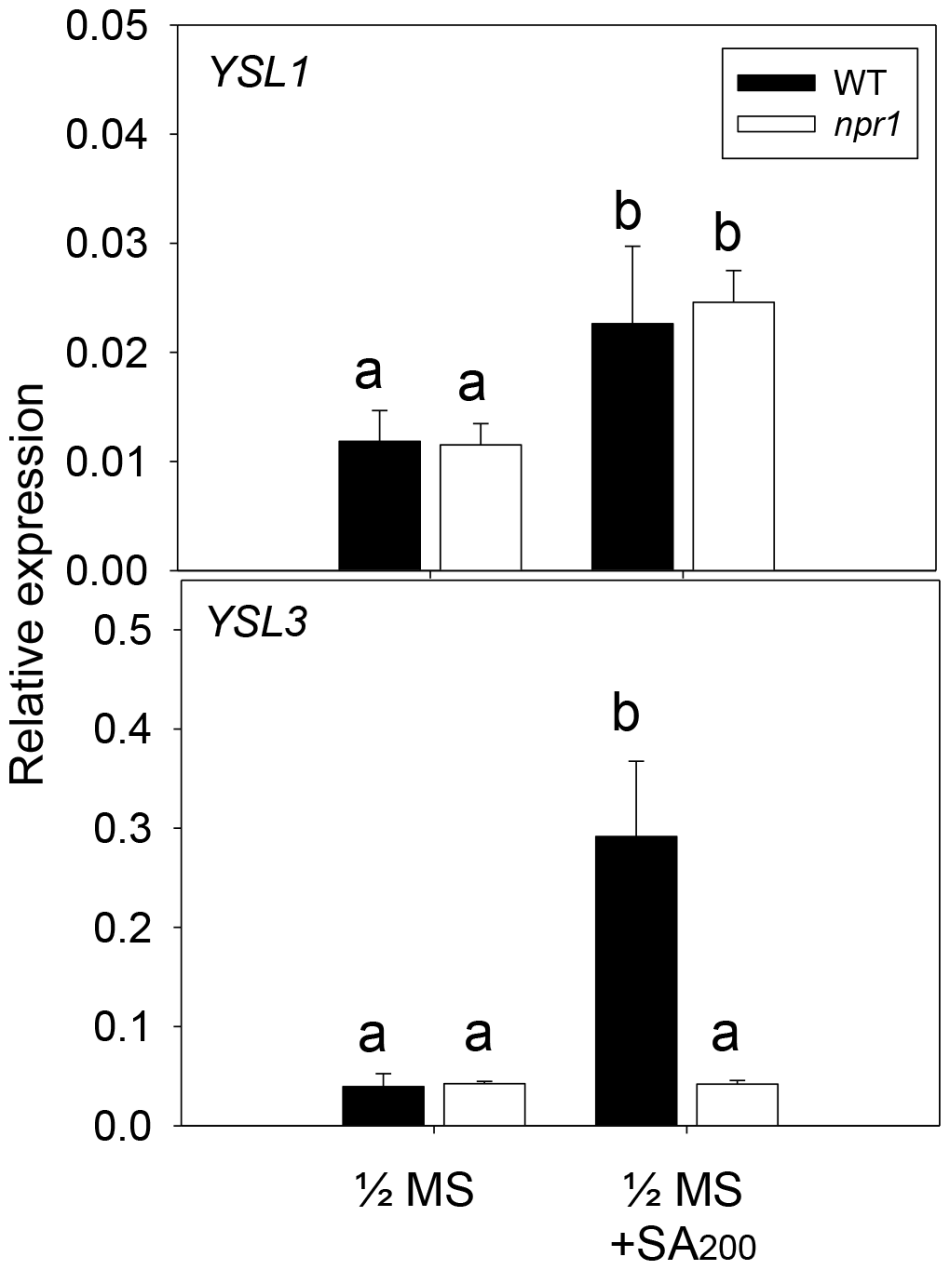
Effect of exogenous SA on the expression of *YSL1* and *YSL3* in *A. thaliana* Col-0 and the *npr1* mutant. Twelve-day-old plants of *A. thaliana* Col-0 wild type (WT) and *npr1* mutant were treated with 200 µM SA for 24 h. qPCR analysis of expression of *YSL1* and *YSL3* in shoots relative to that of *ACT2*. Data are mean±SD 9 samples from 3 biological replicates. Different letters indicate significant difference at p<0.05.

We used wild-type plants treated with SA to investigate whether the expression of *YSL1* and *YSL3* is regulated by SA. *YSL3* was induced by both low and high concentrations of SA in the shoot but only slightly induced by a low concentration in the root (Figure 1). The SA-induced *YSL3* expression was previously observed in whole seedlings [38]. *YSL1* was only slightly induced by a high concentration (200 µM) of SA in the shoot but not in the root. To further address whether SA-induced *YSL1* and *YSL3* expression depends on *NPR1*, we detected the expression of *YSL1* and *YSL3* in the wild type and the *npr1* mutant with 200-µM SA treatment. SA-induced *YSL3* expression was diminished in the *npr1* mutant, so the induction depended on NPR1 (Figure 2). However, SA only slightly induced *YSL1* expression, with no NPR1 dependence (Figure 2). The expression could be induced at 1 h after SA treatment (Figure S1 in File S1). Of note, the induction of *YSL3* expression showed two phases during SA treatment: slightly induced at early times (1 to 8 h) and substantially induced at late times (12 to 24 h).

### The *ysl3* mutant shows enhanced susceptibility against *Pseudomonas syringae* pv. tomato (*Pst*) DC3000

**Figure 3:**
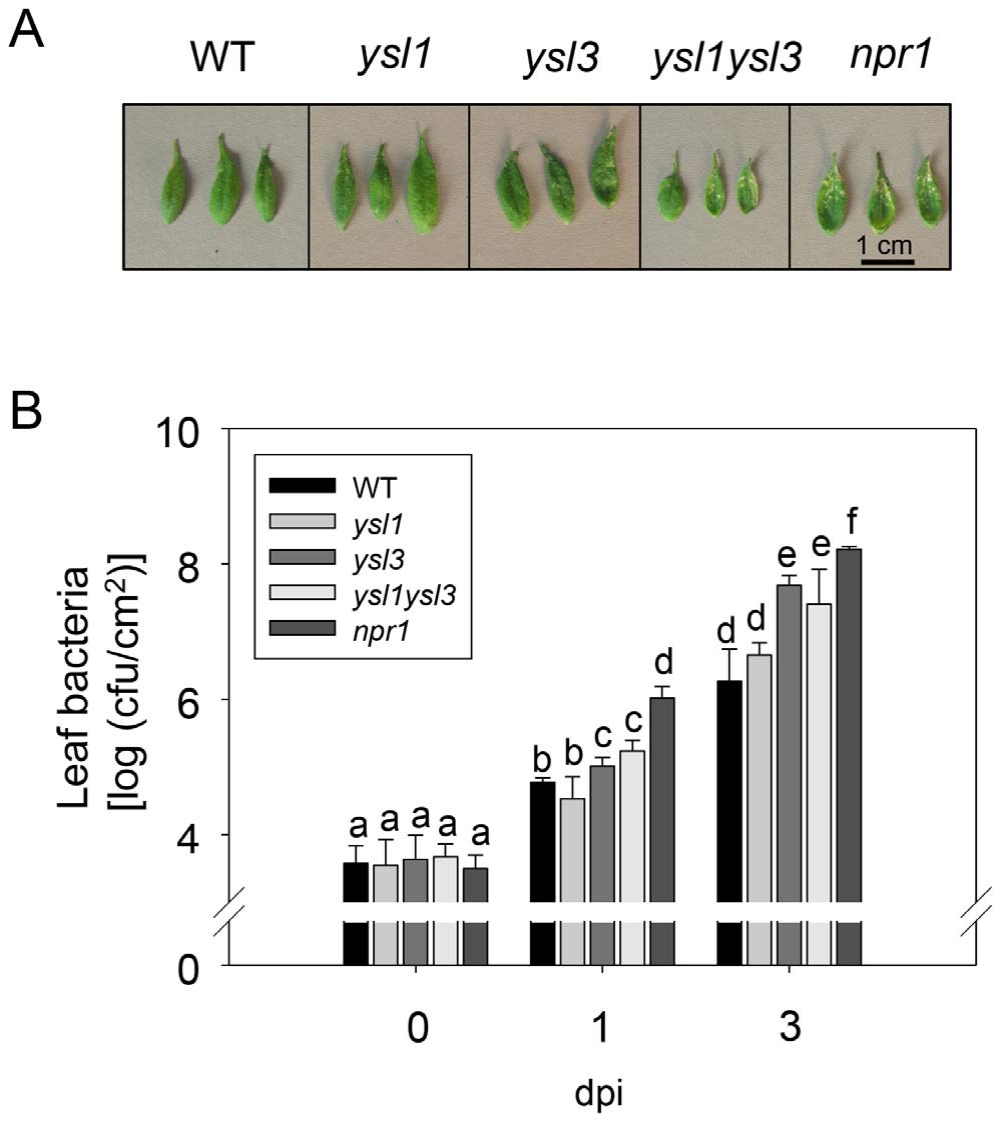
Mutation in *YSL3* results in enhanced susceptibility to *P. syringae* pv. tomato (*Pst*) DC3000 infection. A, Disease symptoms on leaves of each Arabidopsis line after inoculation with *Pst* DC3000. Four-week-old *Arabidopsis thaliana* Col-0 wild type (WT), *ysl1*, *ysl3, ysl1ysl3* and *npr1* grown in the soil were spray-inoculated with 10^7^ colony-forming units (cfu)/mL *Pst* DC3000 in 10 mM MgCl_2_ with 0.02% Silwet L-77 (hereafter *Pst* DC3000 solution). Photographs were taken 3 days post inoculation (dpi). B, Bacterial population in leaves of each Arabidopsis line 0, 1, and 3 days after inoculation. Data are mean±SD from 6 replicates. Different letters indicate significant difference at p<0.05.

SA synthesis and accumulation are required for local and systemic-acquired resistance to pathogen infection. In SA signaling, *NPR1* plays a key role in pathogen defense, because the *npr1* mutant is very sensitive to *Pst* DC3000 infection [39]. SA upregulated *YSL3* expression through *NPR1* (Figure 1, Figure 2) in Arabidopsis; thus, we hypothesized that *YSL3* could be involved in pathogen resistance. Arabidopsis plants of the wild type, *ysl1, ysl3, ysl1ysl3* and *npr1* mutants were challenged with *Pst* DC3000. Arabidopsis defective in *YSL3* was sensitive to *Pst* DC3000. The disease symptoms of *ysl3* and *ysl1ysl3* were similar to that of the *npr1* mutant (Figure 3A). The bacterial number in leaves was higher for *ysl3* and *ysl1ysl3* than the wild type and *ysl1* (Figure 3B). Overall, the bacterial number in plants agreed with the disease symptoms observed. No further change in susceptibility in the defect of *YSL1* in *ysl1* and *ysl1ysl3* mutants suggested that *YSL1* plays no or little role in the plant pathogen defense. The enhanced susceptibility against pathogens in the *ysl3* mutant was further confirmed in the mutant with a second mutated allele (Figures S2, S3 in File S1).

**Figure 4:**
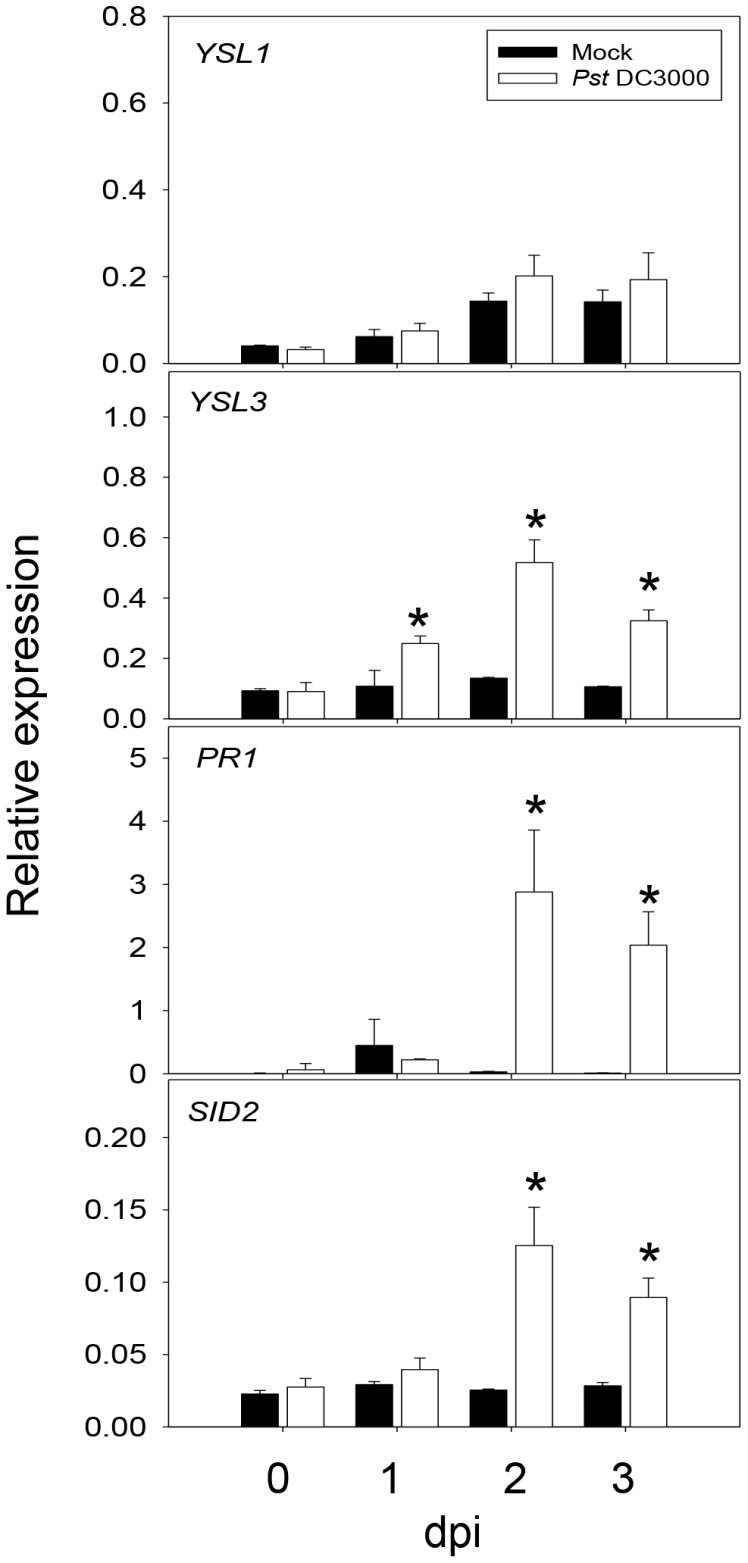
Expression of *YSL1, YSL3*, *PR1* and *SID2* in Arabidopsis leaves infected with *Pst* DC3000. Four-week-old plants of Arabidopsis wild type (Col-0) were spray-inoculated with *Pst* DC3000 solution. Plants sprayed with 10 mM MgCl_2_ with 0.02% Silwet L-77 were used as a mock treatment control. Mature leaves (3rd) were collected 0, 1, 2 and 3 days post-inoculation (dpi). qPCR analysis of expression of *YSL1, YSL3, PR1, SID2* and *PLANT DEFENSIN1.2* (*PDF1.2*) relative to that of *ACT2*. Data are mean±SD from 6 samples of 2 biological repeats. *P<0.05 compared with mock inoculation.

To investigate the induction pattern of *YSL3* in soil-grown plants, we examined the expression of *YSL3* after pathogen infection. *YSL3* was induced immediately after *Pst* DC3000 infection, a pattern similar to that for the SA biosynthesis gene *SALICYLIC ACID INDUCTION DEFICIENT2* (*SID2*) and SA downstream gene *PATHOGENESIS-RELATED1* (*PR1*) (Figure 4). *YSL3* expression was significantly induced after 12-h *Pst* DC3000 inoculation (Figure S4 in File S1). This *Pst* DC3000-induced *YSL3* expression was diminished in *sid2* and *npr1*, similar to the expression of *PR1* (Figure S5 in File S1), which supports a role downstream of SA. Therefore, induction of *YSL3* plays a role in the basal defense against *Pst* DC3000 infection in Arabidopsis.

### *YSL3* is negatively regulated by jasmonic acid (JA) signaling via *CORONATINE INSENSITIVE1 (COI1*) during *Pst* DC3000 infection

Crosstalk between SA and JA allows the plant to activate specific defense responses against a particular invading pathogen [40]. The defense response signaling through SA and JA is often antagonistic. Pathogen-induced accumulation of SA is associated with suppressed JA signaling [41]. Previous studies showed that coronatine (COR), a phytotoxic production by some phytopathogenic *Pseudomonads*, promotes *P. syringae* virulence in Arabidopsis by activating a signaling cascade to suppress SA accumulation and signaling [42]. *Pst* DC3000 exploits the inhibition of SA signaling by JA signaling via activating *COI1* by phytotoxic coronatine [43]–[45]. The *coi1* mutant defective in the JA receptor with enhanced resistance to *Pst* DC3000 is via activation of SA-dependent defense pathway [43], [46]. If *YSL1* and *YSL3* are downstream of SA signaling pathway as shown in Figure 1, we expected that both genes would be higher expression in the *coi1* mutant than wild type after *Pst* Dc3000 infection.

**Figure 5:**
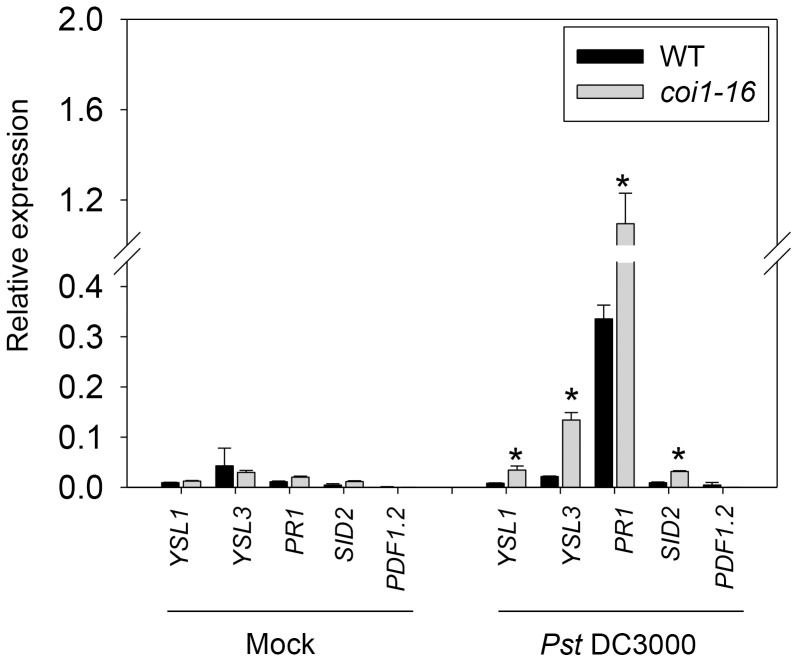
Effect of *CORONATINE INSENSITIVE1* mutation (*coi1*) on the expression of YSL1, *YSL3*, *SID2*, *PR1* and *PDF1.2* in Arabidopsis leaves inoculated with *Pst* DC3000. Four-week-old *A. thaliana* ecotype Col-6 wild type (WT) and *coi1-16* mutant were spray-inoculated with *Pst* DC3000 solution. Plants sprayed with 10 mM MgCl_2_ with 0.02% Silwet L-77 were used as a mock treatment control. Mature leaves (3th) were harvested at 3 dpi. qPCR analysis of expression of *YSL1, YSL3, PR1, SID2 and PDF1.2* relative to that of *ACT2*. Data are mean±SD from 6 samples of 2 biological repeats. *P<0.05 compared with wild type.

We examined gene expression in *coi1* mutant plants spray-inoculated with *Pst* DC3000. In addition to elevated expression of the SA biosynthesis gene *SID2* and SA-regulated *PR1*, the expression of *YSL1* and *YSL3* was elevated in infected *coi1* mutant plants (Figure 5), which supports that *YSL1* and *YSL3* are downstream of SA signaling. By contrast, the expression of *PLANT DEFENSIN1.2* (*PDF1.2*), downstream of JA signaling, was reduced in *coi1* as compared with the wild type (Figure 5). These data suggest that the antagonism between SA and JA signaling applies to the control of *YSL1* and *YSL3.*

In conclusion, we identified that SA-induced *YSL3* expression depends on *NPR1*; *YSL1* is slightly induced by high-concentration SA and *NPR1*-independent. Our results clearly demonstrate the importance of *YSL3* in plant pathogen defense regulated by cross-talk between SA- and JA-dependent signaling pathways during infection with *Pst* DC3000. The knockout mutant *ysl3* was more susceptible than the wild type to *Pst* DC3000 infection. *YSL3* is a positive regulator of basal resistance and appears to function downstream of SA and to be negatively regulated by JA signaling through *COI1*. *SIZ1* is a negative regulator for both the plant defense response [31] and the expression of *YSL3* [33], which may be through the regulation of SA accumulation. Apparently, *YSL3* plays a role in the *SIZ1-*regulated plant defense response.

This regulation information extends our knowledge of the biological function of *YSL3* from metal ion translocation to plant immunity. NPR1 was recently reported to be a receptor of SA, and Cu a potential cofactor for its activation [47]. Whether the metal transporter role of YSL3 contributes to the delivery of Cu for NPR1 activity or *YSL3*-mediated metal ion homeostasis plays a role in SA signaling through ROS in plant pathogen defense remains for further study.

## Supporting Information

File S1 Contains: **Figure S1.** Time-course expression of selected SA-induced genes (SAIG). RT-PCR analysis of selected SAIG gene expression in wild-type plants. Total RNA from 2-week-old seedlings treated with 0.5 mM SA (SA) or maintained in MS medium (C) for the times (hours) indicated were isolated for RT-PCR. *LLP* (At5g03350) and *PR1* (At2g14610) were used as controls for *NPR1*-dependent early and late response genes, respectively. *ACT3* (At3g53750) was a control. The following primers were used for RT-PCR: *YSL3*-FP: 5′-ATGAGGAGTATGATGATGGAGAGAGAG -3′ *YSL3*-RP: 5′-TTAACTCGAATATTTACTCGGCATGAAGC -3′; *LLP*-FP: 5′-TTGGGAAAATGAAACACTGGTC-3′ *LLP*-RP: 5′-CATTCCGGTTACAACTTTCTGATAC-3′; *PR1*-FP, 5′-TTCTTCCCTCGAAAGCTCAA-3′; *PR1*-RP, 5′-TTGCAACTGATTATGGTTCCAC-3′); *ACT3*-FP: 5′-GCTATGTATGTCGCCATTCAAGC-3′ *ACT3*-RP: 5′-CATCATATTCTGCCTTTGCGATCC-3′ Cycles for amplification of each gene are indicated. **Figure S2. Two T-DNA SALK lines, SALK_064683 (** ***ysl3-1*** **) and SALK_045218 (** ***ysl3-2*** **), of** ***YSL3*** **.** A, Relative positions of T-DNA insertions in *YSL3*. White boxes and lines represent exons and introns, respectively. Insertion sites of T-DNA and orientation are illustrated by triangles with arrowheads. Primers used for RT-PCR are indicated. B, Genotyping of *ysl3-1* and *ysl3-2* mutants. Primers used for genotyping are *ysl3-1* LP: CCCTCGATATTTTGCTTAGGG; *ysl3-1* RP: CTTCACCTAGGTCGATGCTTG; *ysl3-2* LP: GCCTTTAGGAGTGTGGAAACC *ysl3-2* RP: TTTTTCCTCTCGTCATTTTCC and LBb1.3: ATTTTGCCGATTTCGGAAC. PCR reactions in 1 involved primers LP and RP and in 2 LBb1.3 and RP. C, RT-PCR to detect the expression of *YSL3*. The following primers were used for RT-PCR: *YSL3* FP: ATGAGGAGTATGATGATGGAGAGAGAG; *YSL3* RP: TTAACTCGAATATTTACTCGGCATGAAGC; *ACT8* FP: CCACATGCTATCCTCCGTCT and *ACT8* RP: CTGGAAAGTGCTGAGGGAAG. *ACT8* (At1g49240) expression was a control. **Figure S3. Two T-DNA insertion lines of** ***ysl3*** **mutants with enhanced susceptibility to** ***P. syringae*** **pv. tomato DC3000 infection.** A, Disease symptoms on leaves of each Arabidopsis line after inoculation with *Pseudomonas syringae* pv. tomato (*Pst*) DC3000. Four-week-old *Arabidopsis thaliana* Col-0 wild type (WT), *ysl3-1*, *ysl3-2* and *npr1* grown in the soil were spray-inoculated with 10^7^ cfu/mL *Pseudomonas syringae* pv. tomato (*Pst*) DC3000 in 10 mM MgCl_2_ with 0.02% Silwet L-77. Photographs were taken 3 days post-inoculation (dpi). Bar scale is 1 cm. B, Bacterial population in leaves of each Arabidopsis line after inoculation of *Pst* DC3000. 0 and 3 dpi, leaf samples were collected and bacterial number was determined. Data are mean±SD from 4 replicates. Different letters indicate significant difference at p<0.05. **Figure S4. Time course of** ***YSL3*** **gene expression in Arabidopsis.** *YSL3* expression in *Pst* DC3000-infected and mock-inoculated (Mock) Arabidopsis (Col-0) plants at 0, 1, 3, 6, 12, 24, 48 and 72 h post-inoculation (hpi) monitored by qPCR. Total RNA from 3-week-old seedlings grown at 22°C on soil were used as templates. qPCR analysis of *YSL3* expression relative to that of *ACT2*. Data are mean±SD from 6 samples of 2 biological repeats. **Figure S5. RT-PCR analysis of** ***YSL3*** **expression in the wild type,** ***sid2*** **and** ***npr1*** **.** RT-PCR analysis of *YSL3* and *PR1* expression in *Pst* DC3000-infected and mock-inoculated (Mock) Arabidopsis Col-0 wild-type (WT), *sid2-1* and *npr1* plants over 2 days post-inoculation (dpi). Total RNA from 3-week-old seedlings grown at 22°C on soil was used as templates. *ACT3* was a control. Cycles for amplification of each gene are indicated.
(PDF)
