# Supplementary material for: Alternative Functions of Arabidopsis YELLOW STRIPE-LIKE3: From Metal Translocation to Pathogen Defense
Source: PLoS One. 2014 May 20;9(5):e98008. doi: 10.1371/journal.pone.0098008 (PMC4028246; doi:10.1371/journal.pone.0098008)
Supplement: File S1 — Contains: Figure S1. Time-course expression of selected SA-induced genes (SAIG). RT-PCR analysis of selected SAIG gene expression in wild-type plants. Total RNA from 2-week-old seedlings treated with 0.5 mM SA (SA) or maintained in MS medium (C) for the times (hours) indicated were isolated for RT-PCR. LLP (At5g03350) and PR1 (At2g14610) were used as controls for NPR1-dependent early and late response genes, respectively. ACT3 (At3g53750) was a control. The following primers were used for RT-PCR: YSL3-FP: 5′-ATGAGGAGTATGATGATGGAGAGAGAG -3′ YSL3-RP: 5′-TTAACTCGAATATTTACTCGGCATGAAGC -3′; LLP-FP: 5′-TTGGGAAAATGAAACACTGGTC-3′ LLP-RP: 5′-CATTCCGGTTACAACTTTCTGATAC-3′; PR1-FP, 5′-TTCTTCCCTCGAAAGCTCAA-3′; PR1-RP, 5′-TTGCAACTGATTATGGTTCCAC-3′); ACT3-FP: 5′-GCTATGTATGTCGCCATTCAAGC-3′ ACT3-RP: 5′-CATCATATTCTGCCTTTGCGATCC-3′ Cycles for amplification of each gene are indicated. Figure S2. Two T-DNA SALK lines, SALK_064683 ( ysl3-1 ) and SALK_045218 ( ysl3-2 ), of YSL3 . A, Relative positions of T-DNA insertions in YSL3. White boxes and lines represent exons and introns, respectively. Insertion sites of T-DNA and orientation are illustrated by triangles with arrowheads. Primers used for RT-PCR are indicated. B, Genotyping of ysl3-1 and ysl3-2 mutants. Primers used for genotyping are ysl3-1 LP: CCCTCGATATTTTGCTTAGGG; ysl3-1 RP: CTTCACCTAGGTCGATGCTTG; ysl3-2 LP: GCCTTTAGGAGTGTGGAAACC ysl3-2 RP: TTTTTCCTCTCGTCATTTTCC and LBb1.3: ATTTTGCCGATTTCGGAAC. PCR reactions in 1 involved primers LP and RP and in 2 LBb1.3 and RP. C, RT-PCR to detect the expression of YSL3. The following primers were used for RT-PCR: YSL3 FP: ATGAGGAGTATGATGATGGAGAGAGAG; YSL3 RP: TTAACTCGAATATTTACTCGGCATGAAGC; ACT8 FP: CCACATGCTATCCTCCGTCT and ACT8 RP: CTGGAAAGTGCTGAGGGAAG. ACT8 (At1g49240) expression was a control. Figure S3. Two T-DNA insertion lines of ysl3 mutants with enhanced susceptibility to P. syringae pv. tomato DC3000 infection. A, Disease symptoms on leaves of each Arabidopsis line after inoculation [file pone.0098008.s001.pdf]

Figure S1.

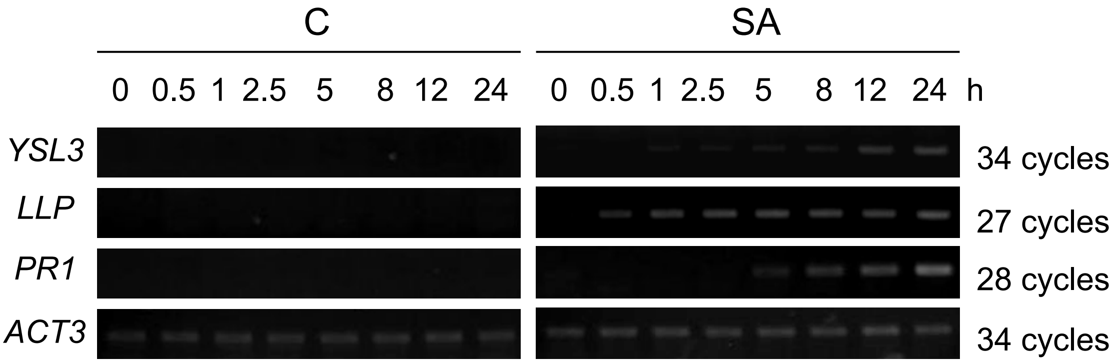

Figure S1. Time-course expression of selected SA-induced genes (SAIG). RT-PCR analysis of selected SAIG gene expression in wild-type plants. Total RNA from 2-week-old seedlings treated with 0.5 mM SA (SA) or maintained in MS medium (C) for the times (hours) indicated were isolated for RT-PCR. *LLP* (At5g03350) and *PR1* (At2g14610) were used as controls for *NPR1*-dependent early and late response genes, respectively. *ACT3* (At3g53750) was a control. The following primers were used for RT-PCR:

YSL3-FP: 5'-ATGAGGAGTATGATGATGGAGAGAGAG -3'
YSL3-RP: 5'-TTAACTCGAATATTTACTCGGCATGAAGC -3';
LLP-FP: 5'-TTGGGAAAATGAAACACTGGTC-3'
LLP-RP: 5'-CATTCCGGTTACAACTTTCTGATAC-3';
PR1-FP, 5'-TTCTTCCCTCGAAAGCTCAA-3';
PR1-RP, 5'-TTGCAACTGATTATGGTTCCAC-3');
ACT3-FP: 5'-GCTATGTATGTCGCCATTCAAGC-3'
ACT3-RP: 5'-CATCATATTCTGCCTTTGCGATCC-3'

Figure S2.

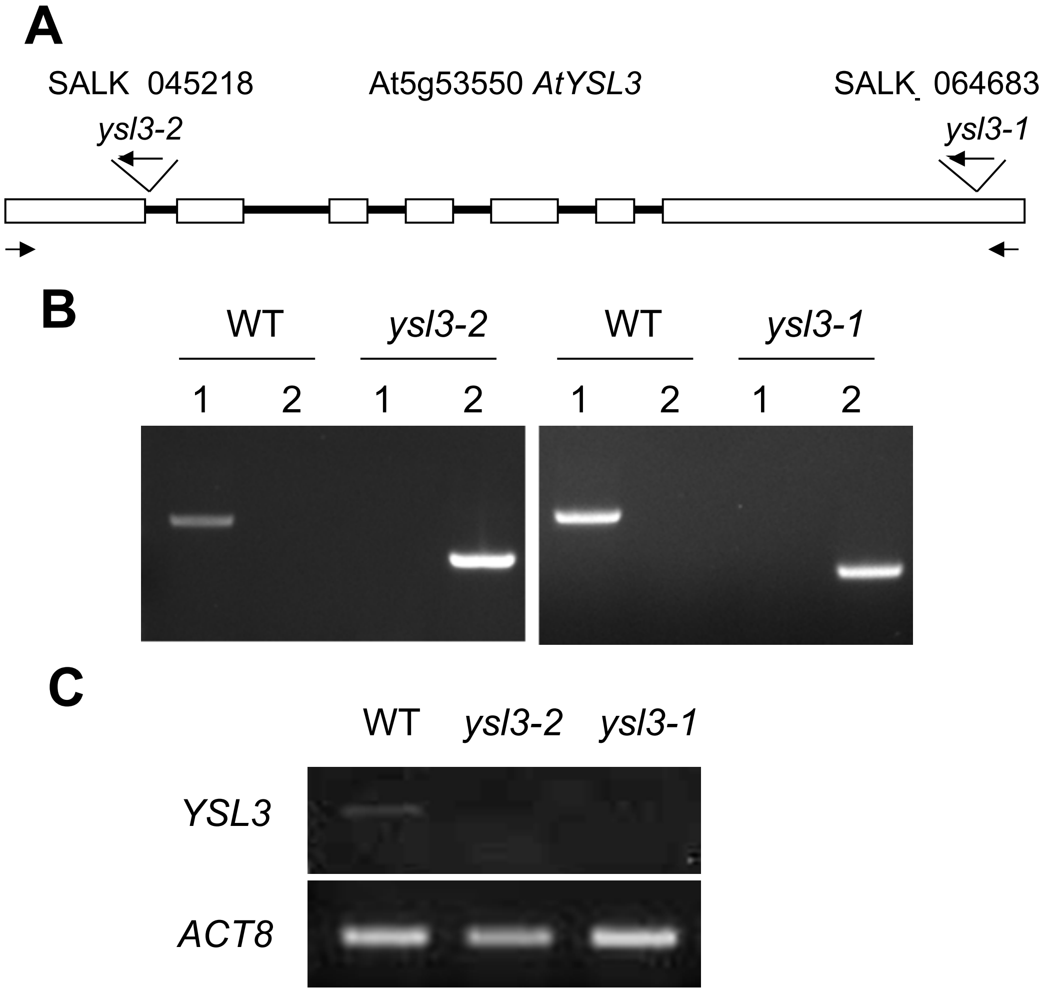

Figure S2. Two T-DNA SALK lines, SALK_064683 (*ysl3-1*) and SALK_045218 (*ysl3-2*), of *YSL3*. A, Relative positions of T-DNA insertions in *YSL3*. White boxes and lines represent exons and introns, respectively. Insertion sites of T-DNA and orientation are illustrated by triangles with arrowheads. Primers used for RT-PCR are indicated. B, Genotyping of *ysl3-1* and *ysl3-2* mutants. Primers used for genotyping are *ysl3-1* LP: CCCTCGATATTTTGCTTAGGG; *ysl3-1* RP: CTTCACCTAGGTCGATGCTTG; *ysl3-2* LP: GCCTTTAGGAGTGTGGAAACC; *ysl3-2* RP: TTTTTCCTCTCGTCATTTTCC and LBb1.3: ATTTTGCCGATTTCGGAAC. PCR reactions in 1 involved primers LP and RP and in 2 LBb1.3 and RP. C, RT-PCR to detect the expression of *YSL3.* The following primers were used for RT-PCR: *YSL3* FP: ATGAGGAGTATGATGATGGAGAGAGAG;
YSL3 RP: TTAACTCGAATATTTACTCGGCATGAAGC;
ACT8 FP: CCACATGCTATCCTCCGTCT and *ACT8* RP: CTGGAAAGTGCTGAGGGAAG. *ACT8* (At1g49240) expression was a control.

Figure S3.

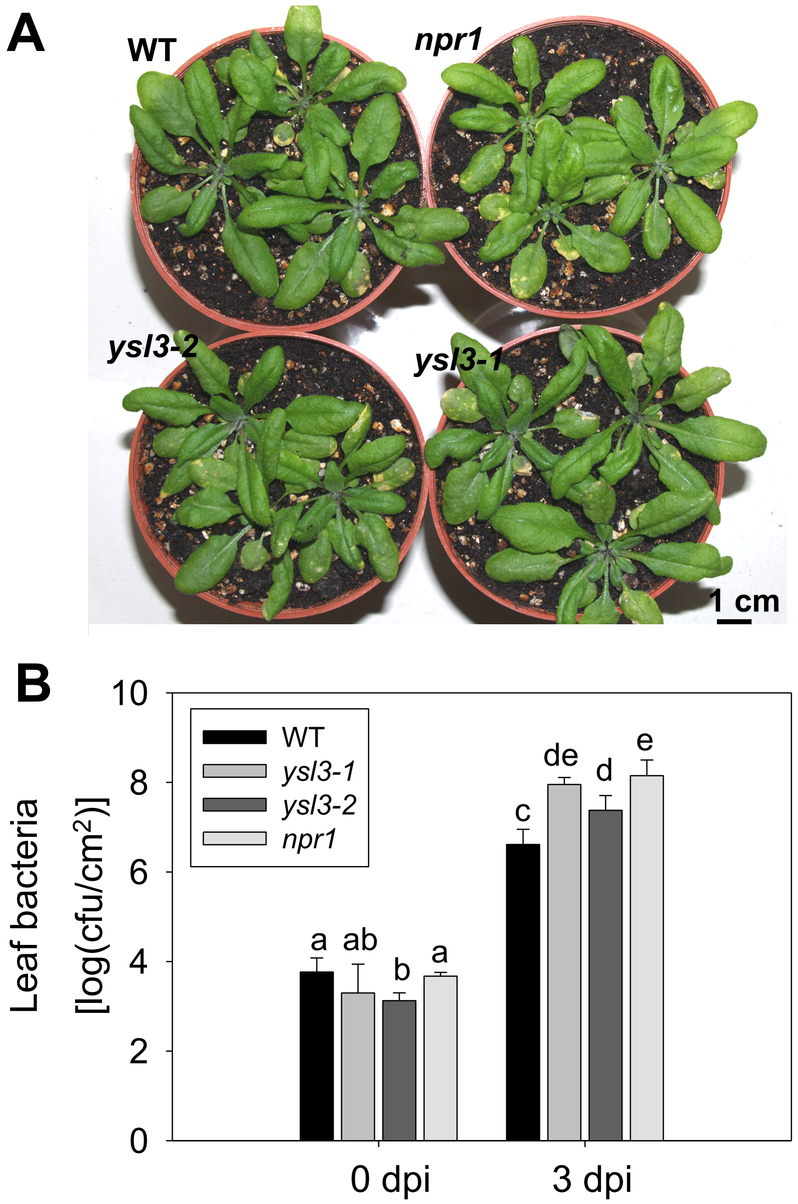

Figure S3. Two T-DNA insertion lines of *ysl3* mutants with enhanced susceptibility to *P. syringae* pv. tomato DC3000 infection. A, Disease symptoms on leaves of each Arabidopsis line after inoculation with *Pseudomonas syringae* pv. tomato (*Pst*) DC3000. Four-week-old *Arabidopsis thaliana* Col-0 wild type (WT), *ysl3-1*, *ysl3-2* and *npr1* grown in the soil were spray-inoculated with 10^7 cfu/mL *Pseudomonas syringae* pv. tomato (*Pst*) DC3000 in 10 mM $MgCl_2$ with 0.02% Silwet L-77. Photographs were taken 3 days post-inoculation (dpi). Bar scale is 1 cm. B, Bacterial population in leaves of each Arabidopsis line after inoculation of *Pst* DC3000. 0 and 3 dpi, leaf samples were collected and bacterial number was determined. Data are mean ±SD from 4 replicates. Different letters indicate significant difference at $p < 0.05$.

Figure S4.

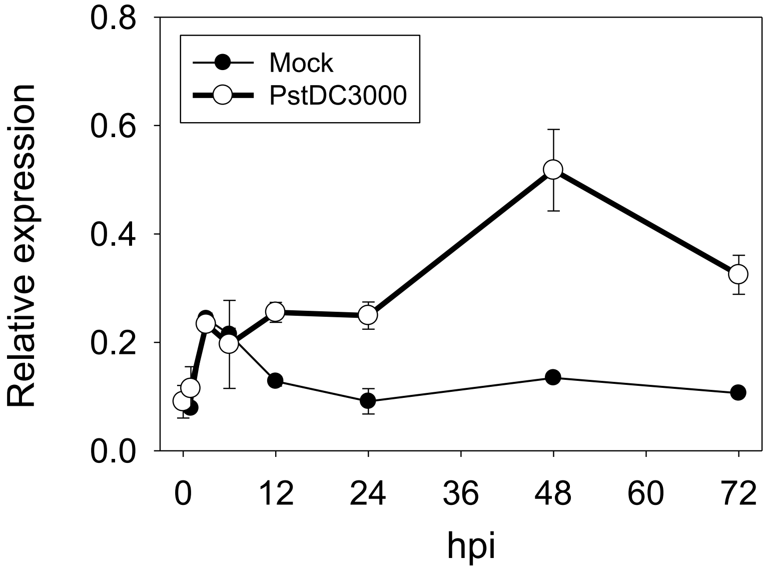

Figure S4. Time course of *YSL3* gene expression in Arabidopsis. *YSL3* expression in *Pst* DC3000-infected and mock-inoculated (Mock) Arabidopsis (Col-0) plants at 0, 1, 3, 6, 12, 24, 48 and 72 h post-inoculation (hpi) monitored by qPCR. Total RNA from 3-week-old seedlings grown at 22°C on soil were used as templates. qPCR analysis of *YSL3* expression relative to that of *ACT2*. Data are mean ±SD from 6 samples of 2 biological repeats.

Figure S5.

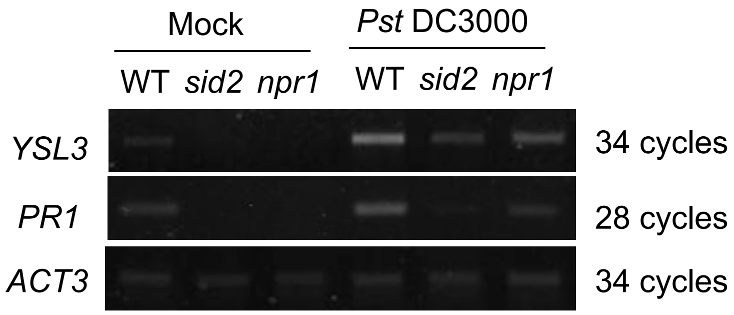

Figure S5. RT-PCR analysis of *YSL3* expression in the wild type, *sid2* and *npr1*. RT-PCR analysis of *YSL3* and *PR1* expression in *Pst* DC3000-infected and mock-inoculated (Mock) Arabidopsis Col-0 wild-type (WT), *sid2-1* and *npr1* plants over 2 days post-inoculation (dpi). Total RNA from 3-week-old seedlings grown at 22°C on soil was used as templates. *ACT3* was a control. Cycles for amplification of each gene are indicated.
